# Endodontic and Clinical Considerations in the Management of Variable Anatomy in Mandibular Premolars: A Literature Review

**DOI:** 10.1155/2014/512574

**Published:** 2014-05-08

**Authors:** Denzil Albuquerque, Jojo Kottoor, Mohammad Hammo

**Affiliations:** ^1^Endodontic Speciality Practice, Orlem, Malad, Mumbai 400064, India; ^2^Department of Conservative Dentistry and Endodontics, Mar Baselios Dental College, Kothamangalam, Ernakulam, Kerala 686691, India; ^3^Hammo Endodontic Speciality Clinic, Amman 11110, Jordan

## Abstract

Mandibular premolars are known to have numerous anatomic variations of their roots and root canals, which are a challenge to treat endodontically. The paper reviews literature to detail the various clinically relevant anatomic considerations with detailed techniques and methods to successfully manage these anomalies. An emphasis and detailed description of every step of treatment including preoperative diagnosis, intraoperative identification and management, and surgical endodontic considerations for the successful management of these complex cases have been included.

## 1. Introduction

Endodontics is the branch of dentistry which is concerned with the morphology, physiology, and pathology of the human dental pulp and periradicular tissues. Its study and practice encompass the basic and clinical sciences including biology of the normal pulp and the etiology, diagnosis, prevention, and treatment of diseases and injuries of the pulp and associated periradicular conditions [1]. Endodontic management of a tooth is an interesting and challenging procedure that is partly unsighted, and its success depends on individual clinical acumen and skill, as well as the procedural techniques applied. Tooth anatomy is the blueprint on which every clinician relies upon prior to initiating root canal treatment. The vast data on tooth anatomy available to the scientific community does allow for a better understanding of the tooth's internal anatomy and its numerous variations. However, this very literature also lacks direct and categorical inferences to any geographic or ethnic patterns. In a recently published systematic literature review on root anatomy and canal configuration of permanent mandibular premolars, direct evidence to correlate certain anatomic variations in mandibular premolars to specific ethnic and geographic populations was reported [2].

Clinically, how does the endodontist utilize this information regarding tooth anatomy to enhance efficacy in root canal treatment? Awareness of the probable variations in root and root canal anatomy reduces the possibility of missed canals during treatment. Also, it inculcates an outlook, wherein the tooth in question is approached with a view that an anomalous root or root canal is a certainty rather than a rarity. However, this is just the first step towards reliable endodontics. Once the anomaly has been identified preoperatively, it is an added challenge to negotiate the variations by identifying them intra-operatively, followed by cleaning, shaping and obturating the same.

Mandibular premolars have on multiple occasions been stated to be the most challenging teeth to be treated endodontically, especially when they present with multiple roots or canals [3, 4]. Their propensities for anomalous variations, narrow mesiodistal dimensions and the ensuing narrow access to canals, lack of visibility, and apical third trifurcations and deltas are factors that further compound the difficulty for clinicians. The purpose of this paper is to outline relevant documented clinical methods and techniques that would be beneficial to successfully negotiate various anatomic aberrations specific to mandibular premolars, that is, preoperative identification, root canal access opening, intraoperative identification, location, instrumentation, debridement, disinfection, obturation, and surgical endodontics.

## 2. Methodology

An exhaustive search was undertaken to identify associated literature of mandibular premolars through MEDLINE/PubMed database using keywords “root canal anatomy,” “root canal morphology,” “mandibular premolars,” “mandibular first premolar,” and “mandibular second premolar,” alone or in combination. Documented case reports of variations in mandibular premolar anatomy were identified and a systematic review of the relevant articles was performed for articles dated May 2013 and before. Different clinical techniques applied to manage the endodontic variations of mandibular premolars were summarized under the following headings:

1. identification,
2. endodontic management,
3. surgical management.

## 3. Results and Discussion

### 3.1. Identification of Aberrant Premolar Morphology 

#### 3.1.1. Epidemiological Data

A recently published systematic literature review by Kottoor et al. [2] summarized that lower bicuspids were commonly found to have a single root (greater than 97%) with the mandibular first premolars being comparatively twice more likely to present with two canals (23.55%) than second premolars (12.64%). Multiplicities of roots (up to 4 roots) and root canals (up to 5 canals) have been documented as case reports in both lower bicuspids. In first premolars, C-shaped canals showed a higher incidence in Mongoloids (up to 24%) while other common variations of the first premolar included a deep mesial radicular invagination (13–27%) and dens invaginatus. The review revealed a statistically significant intraethnic and interethnic correlation among Caucasian, Indian, Mongoloid, and Middle Eastern ethnicities, in terms of number of roots, root canals, and apical foramen of mandibular bicuspids. Hence when treating patients from these ethnicities, the clinician should consider the possible anatomic variations that can occur [2]. It is beyond the scope of this paper to further review in depth the numerous anatomic variations of mandibular premolars, and further reading of the systematic review of aberrant mandibular premolar anatomy [2] is highly suggested for a detailed analysis.

#### 3.1.2. Clinical

**Figure 1:**
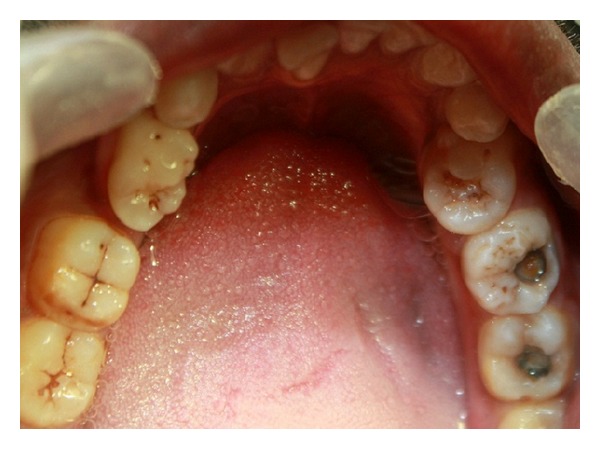
Occlusal photograph showing bilateral premolar molarization in the mandibular second premolars (reprinted with permission from Rajesh Ebenezar et al. [6]).

Thorough preoperative clinical assessment is the first step in trying to unfold any external or internal radicular aberrations. Meticulous observation of the coronal and radicular anatomy and landmarks are prerequisites for the detection of variable anatomy (Figure 1). Atypical crown dimensions, additional cusps, tubercles, deep longitudinal developmental grooves, and fusion of teeth all point to anomalous tooth morphology, which could be accompanied by anomalous endodontic morphology. In case of a carious tooth that gives little information regarding the coronal anatomy, observing the anatomy of the contralateral tooth could give vital insights into the possible underlying anatomy. Gingival recession may reflect the furcal morphology in multirooted teeth and hint at the presence of two buccal roots. Probing the buccal sulcus to feel the root eminences and furcal anatomy may also help to identify the presence of two buccal roots, if present [5].

#### 3.1.3. Radiography

**Figure 2:**
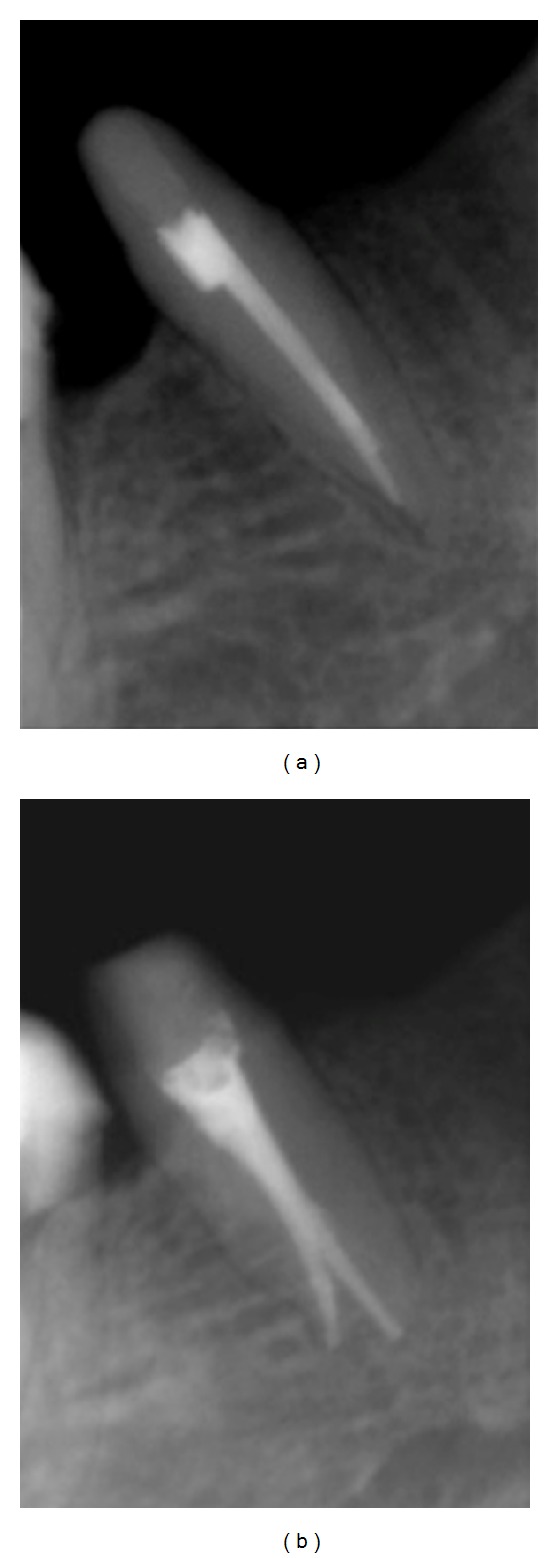
(a) Radiograph in straight angulation. (b) An additional radiograph with a 15–20 degrees horizontal angulation clearly showing a dividing canal.

**Figure 3:**
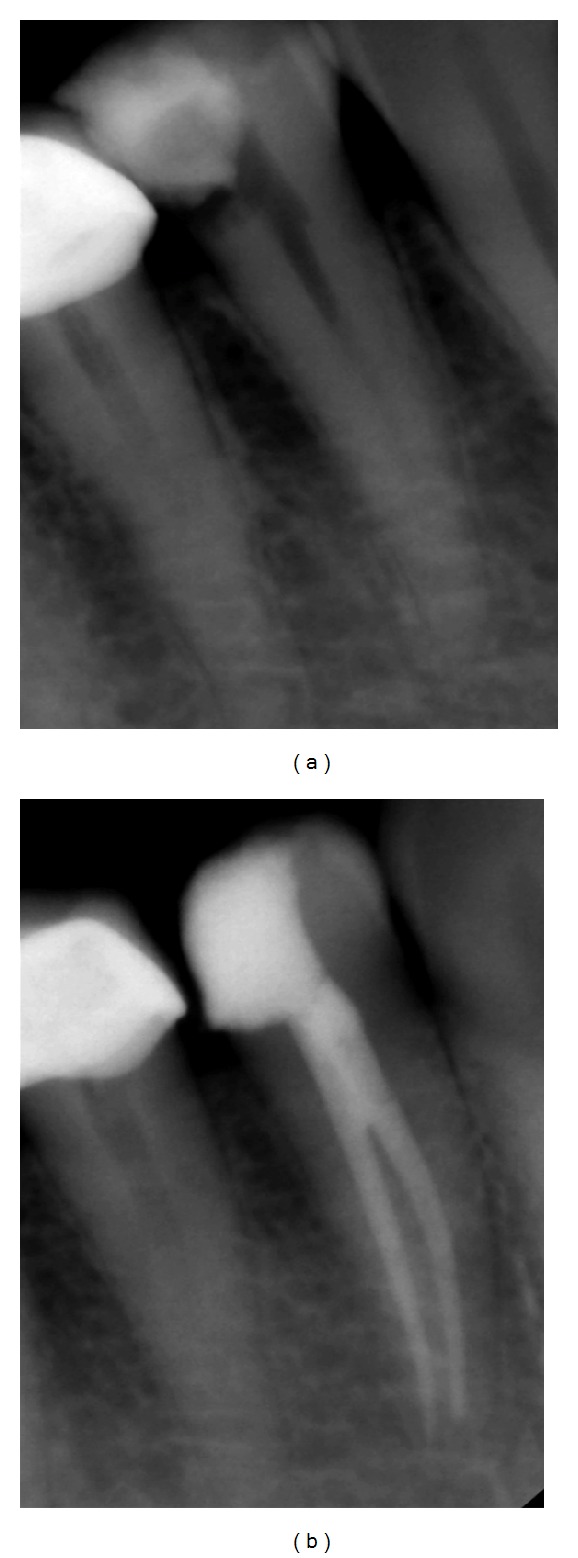
(a) A sudden change in radiographic density at the coronal third indicates canal bifurcation, which is distinctly visible on an angulated radiograph (b).

Radiographs provide much needed information about root canal morphology. Accurate multiple preoperative radiographs, straight and angled, using the paralleling technique are essential to provide evidences about the number of roots. A second radiograph 15 to 20 degrees mesial or distal to the horizontal long axis of the root, is useful to accurately diagnose the number of roots and canals in premolar teeth (Figure 2). A sudden change in radiographic density of the root canal space usually indicates an additional canal or a canal bifurcation [4]. If a radiograph shows a sudden narrowing or even a disappearing pulp space, the canal diverges at that point into two parts that may either remain separate or merge before reaching the apex [7] (Figure 3). Martínez-Lozano et al. [8] have suggested a 40-degree mesial angulation of the X-ray beam to identify additional canals.

**Figure 4:**
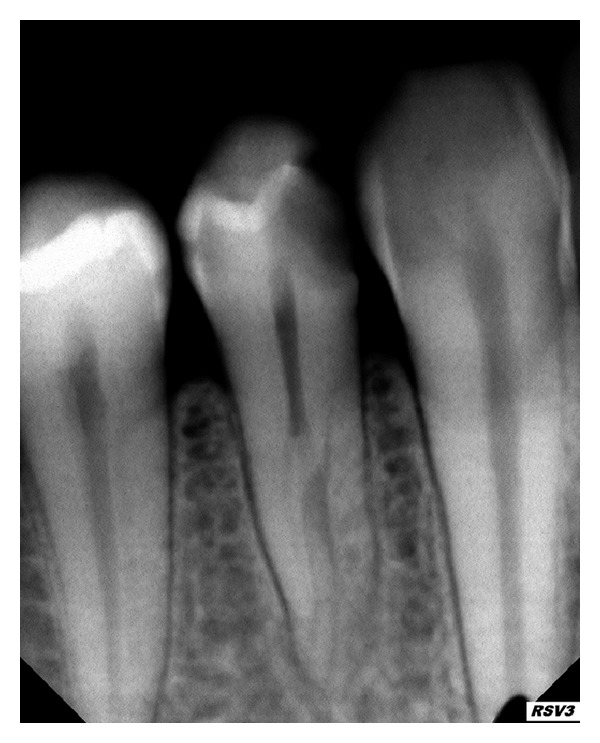
Two periodontal ligament spaces on one side of a root (mesially) and a sudden change in the pulpal radio-density point to multiple roots and canals, respectively.

In mandibular premolars with three canals, the cervical half of the root is generally wider than usual, with little or no taper [5]. Two periodontal ligament spaces on one side of a root and the periodontal ligament space crossing over roots are important observations to indicate the existence of accessory roots (Figure 4). A magnifying device or loupes used during interpretation of radiographs could allow for visualization of some unseen details [7].

**Figure 5:**
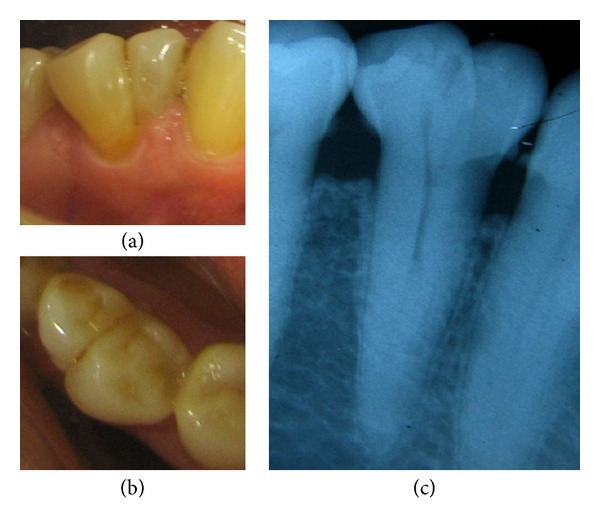
Photographs (a and b) and multiple angulated radiographs (c) of a geminated mandibular first premolar showing the complexity of the internal and external and coronal and radicular anatomy. Multiple canals that fuse and redivide add to the endodontic challenge.

**Figure 6:**
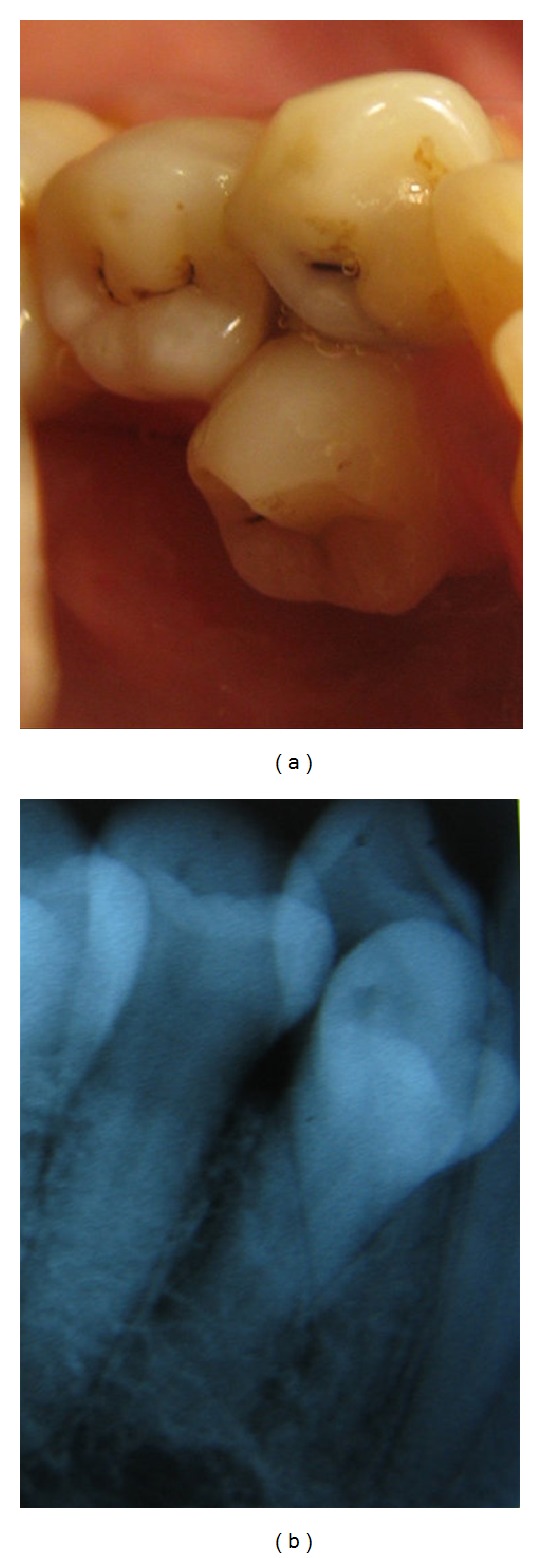
Clinical (a) and radiographic (b) images of a supernumerary fully formed lower bicuspid with an exaggerated linguoocclusal inclination that could add to the endodontic challenge of visibility, access, and instrumentation.

Additional developmental anatomic variations of mandibular premolars include fusion or gemination (Figure 5), fusion with adjacent or supernumerary teeth, dens invaginatus, dens evaginatus, taurodontism, C-shaped canals, and supernumerary premolars (Figure 6). Each of these developmental anomalies is peculiar in the way it presents itself and no two teeth with the same anomaly may be similar. Thus, a mandibular premolar with a developmental anomaly should be thoroughly assessed, clinically and radiographically, to determine the type and extent of the variation. Such aberrant teeth need to be treated as per the individual variation presented.

#### 3.1.4. Advanced Radiography

**Figure 7:**
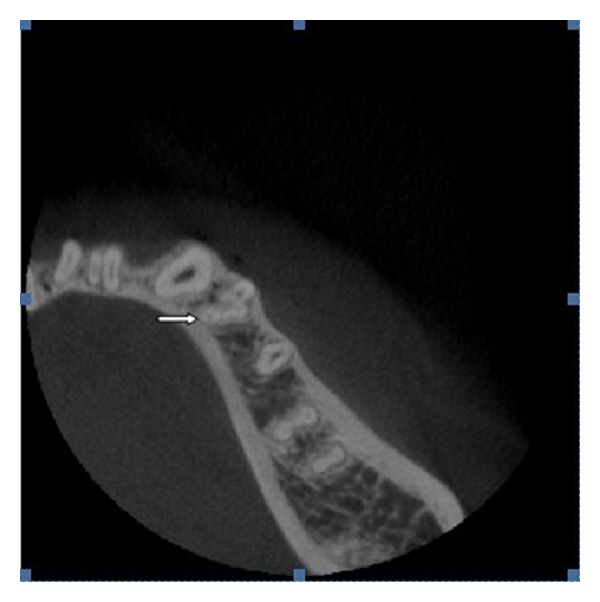
CBCT cross-sectional image of a mandibular first premolar (arrow), showing the spatial relation of a type VIII canal configuration and a deep mesial radicular groove (reprinted with permission from Yu et al. [11]).

CBCT is also a useful tool for the preoperative diagnosis of complex root canal anatomy [9]. The sagittal, coronal, and axial CBCT images provide three-dimensional imaging with relatively lower effective radiation doses than other computed tomography (CT) systems. CBCT also eliminates the problem of superimposition of roots by surrounding anatomical structures. CBCT images provide an insight into the spatial relation of the anatomic variations and allow the clinician to visualize any access modifications that could be required for treatment (Figure 7). Remaining dentin thickness could be measured to avoid overthinning of the dentinal walls or strip perforations in narrow premolar roots. CBCT images can also show some misleading findings [10] and should be interpreted judiciously in conjunction with multiple periapical radiographs. Hence, the final diagnosis should be based on the collective interpretation of clinical and radiographic data.

#### 3.1.5. Intraoperative****

**Figure 8:**
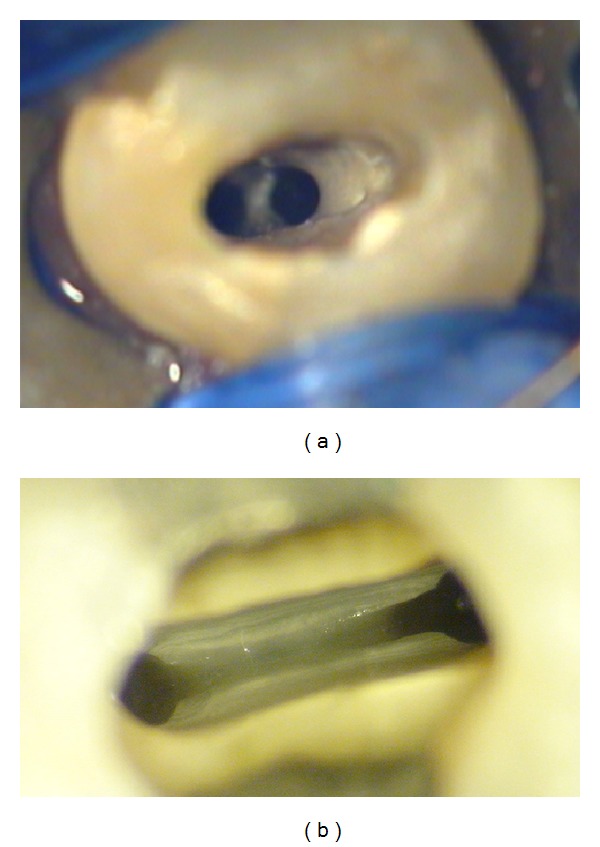
Modification of the access cavity in a mandibular premolar to a buccolingually directed oval access for identification and instrumentation of the second canal.

*(i) Access Cavity.* Detection and management of the entire root canal system in mandibular premolars are extremely challenging, and optimum access cavity preparation is an absolute necessity. In single-canalled lower premolars, the access can be somewhat more circular in nature. However, if a second canal (or bifurcation) is suspected, a buccolingually directed oval access cavity is more appropriate (Figure 8). If the pulp chamber appears to deviate from normal configuration and seems to be either triangular in shape or too large in a mesiodistal plane, more than one root canal should be suspected [12].

**Figure 9:**
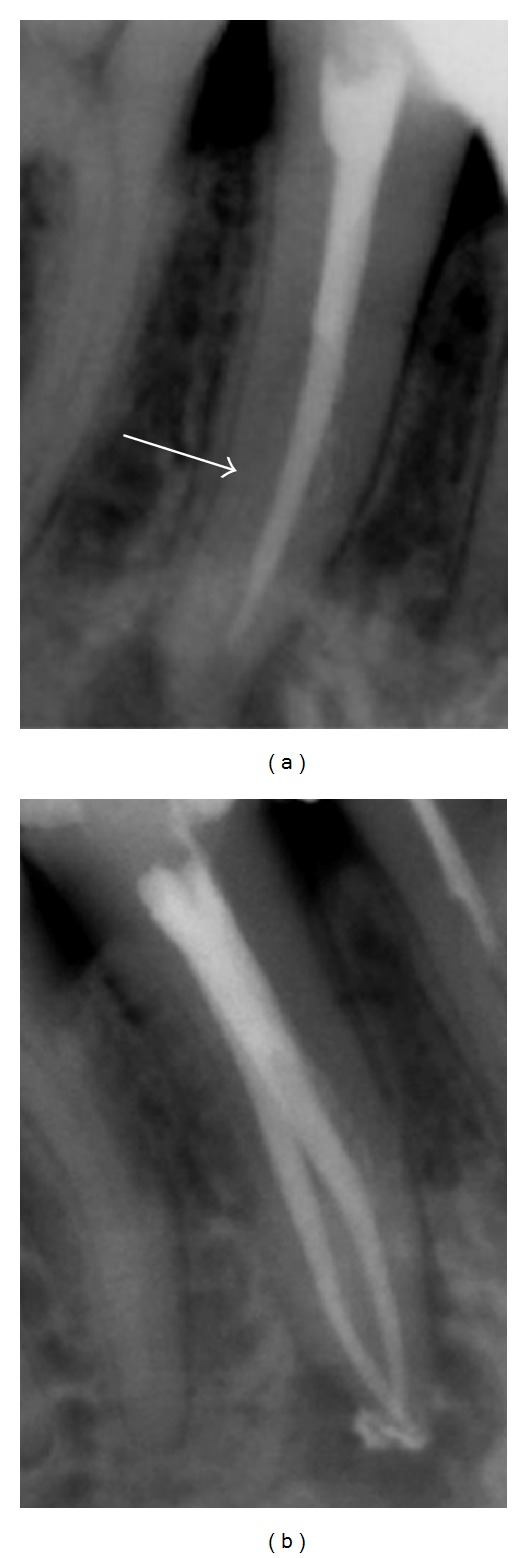
(a) A mandibular second premolar with a missed lingual canal seen distal to the obturated straight buccal canal (white arrow), seen as a mid-root off-shoot at a sharp angle, visualized as a lower letter case “h.” (b) Postretreatment radiograph with obturation of both buccal and lingual canals.

When one canal bifurcates into two, the division is most probably buccal and lingual, with the lingual canal generally splitting from the main canal at a sharp angle, sometimes at nearly the right angle. In addition, the two canals and bifurcations are predominantly at the middle/apical third of the root [13]. So, it is a challenge to determine the exact configuration of the canal system by visual inspection of the pulp chamber floor. Hence, the clinician should make every effort to negotiate the second canal at the middle/apical third. Slowey [4] recommends that it is helpful to visualize this canal configuration as a lower case letter “h.” The buccal canal would be the straight-line portion of the letter “h,” whereas the lingual canal exists about mid-root at a sharp angle from the straight buccal canal (Figure 9). In addition, the lingual inclination of the crown tends to direct instruments buccally, increasing the difficulty of locating the lingual canal orifice. To overcome this situation, the clinician may need to extend the lingual wall of the access cavity more towards the lingual, in order to achieve unobstructed passage of instruments into the lingual canal.

Many complicating factors make the C-shaped configuration of the mandibular first premolar difficult to treat, especially in comparison to mandibular second molars. The C-shaped morphology in mandibular second molars is mostly found coronally and up to 3 mm apical to the cement-enamel junction. Thus, in mandibular second molars, the C-shaped morphology would be more readily appreciated on observation of the floor of the pulp chamber. On the other hand, in the mandibular premolars, coronally, it is a single oval canal and the C-shaped anatomy is located at the apical 3 mm and/or 6 mm level cross-sections, making identification of C-shaped anatomy in mandibular premolars more challenging [14, 15]. Anatomically, the diameter and width of mandibular first premolar are much smaller than mandibular second molars further limiting coronal access to the complex apical root canal system. Thus, C-shaped canals would be difficult to detect from the coronal approach reinforcing the importance of coronal enlargement, enhanced illumination, and magnification. 

**Figure 10:**
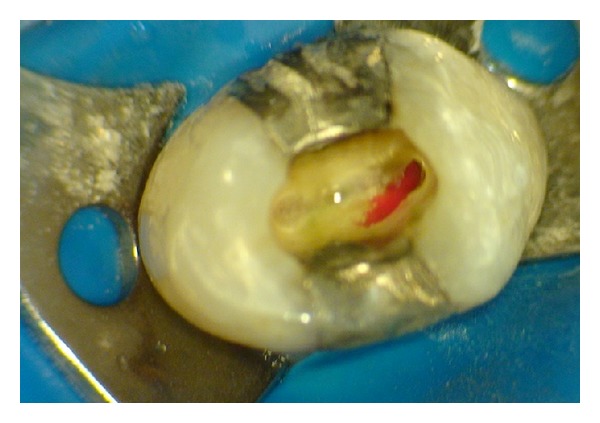
Eccentric bleeding points indicating the presence and location of the buccal canal.

*(ii) Root Canal Exploration. *Means of magnification (ocular loops) and additional lighting (fibre optic illumination) are recommended. Additionally, examination of the pulp chamber floor with a sharp explorer (DG-16/CK-17), troughing of grooves with ultrasonic tips, staining the chamber floor with 1% methylene blue dye or ophthalmic dyes, performing the sodium hypochlorite “champagne bubble” test, and visualizing bleeding points are important aids in locating root canal orifices [16]. If only one eccentric orifice can be found, at least one more canal is present and should be searched for in the opposite side [17] (Figure 10).

The distance between two root canal orifices of mandibular first premolars with two root canals is mostly within 1–3 mm; that is, when 1 root canal orifice has been probed, another root canal orifice can be probed within 1–3 mm. However, wider distances (up to 5 mm) have also been noted between canal orifices [18].

The root canal system of premolars with two roots and three root canals is usually characterized by one large lingual canal and two smaller mesiobuccal and distobuccal canals in the buccal root. A third canal should be suspected clinically when the pulp chamber does not appear to be aligned in its expected buccal-lingual relationship [19]. According to Rödig and Hülsmann [19], the characteristic of mandibular premolars with three root canals is the presence of a triangle-shaped pulp chamber in which the distance from the mesiobuccal to the lingual orifices was the highest. It is suggested that this geometric configuration could help in the latter localization of the DB canal. Recently, Ordinola-Zapata et al. [18] assessed the morphology of mandibular premolars with three root canals using microcomputed tomography and concluded that triangle-shaped configuration of the orifices was more prevalent. A linear configuration could also be encountered very rarely. Tzanetakis et al. [20] suggested the use of symmetry laws proposed by Krasner and Rankow [21], especially when an unexpected or unusual anatomy is present.

**Figure 11:**
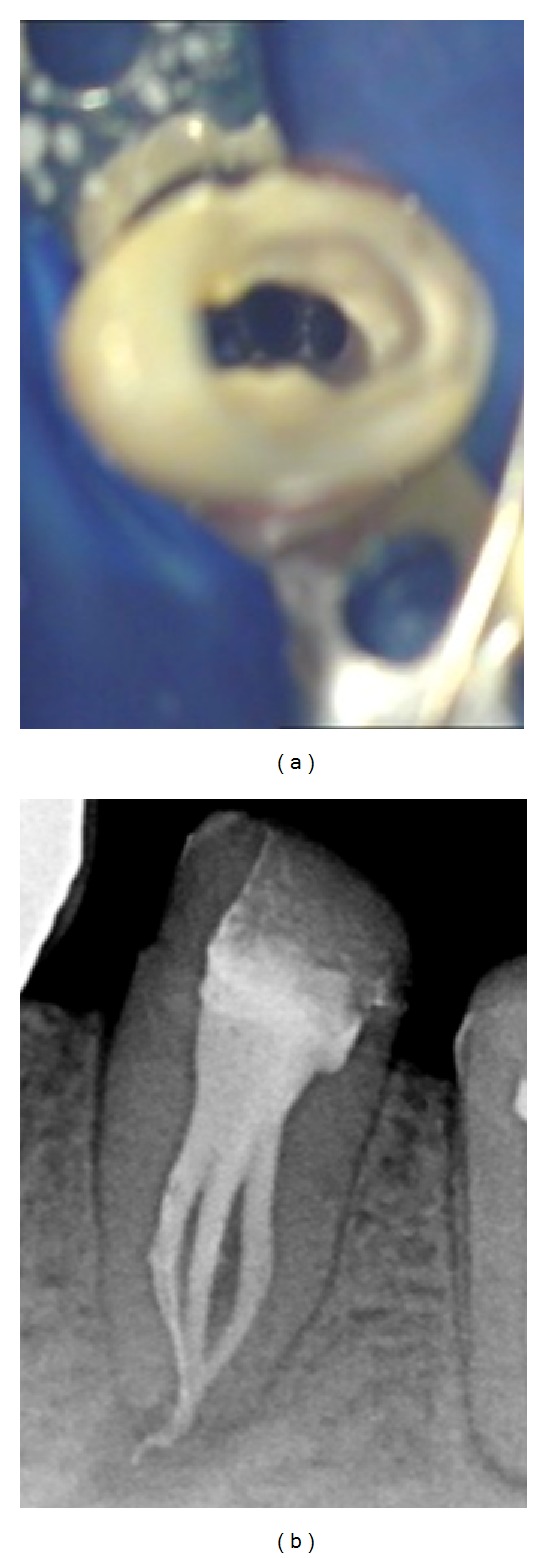
(a) Operating microscopic view showing the clinical trifurcation and the orientation of canal orifices. (b) Postobturation radiograph.

The role of microscopy in endodontics should not be understated. The dental operating microscope should be used to facilitate the observation of anatomical landmarks in the pulp chamber floor that may help to identify supplementary root canals or root canal aberrations [12]. Furthermore, the operating microscope can often enable clinicians to directly visualize the point where the main canal bi- or trifurcates and the orientation of canal orifices (Figure 11). However, if the level of the furcation is deep and canal orifices are calcified, their identification may be difficult, even with a microscope.

Tactile examination of all the walls of the major canal with the tip of a small, precurved scouting K-file is recommended, in order to probe for a catch, which may indicate the orifice of an additional canal. The presence of an additional canal should be suspected whenever an instrument demonstrates an eccentric direction on deeper penetration into the canal, termed directional control, as reported by Green [22]. Under the microscope, identification and initial instrumentation of an additional canal are aided by fine, hand held instruments like the Micro-Orifice Opener (Maillefer, Dentsply).

### 3.2. Endodontic Management of Aberrant Premolar Morphology

#### 3.2.1. Working Length

Radiographic interpretation of some accessory roots and their root apices may be difficult due to their close proximity to each other, and also there exists a possibility of superimposition by other roots. An apex locator is very useful in such cases and can be used to estimate the root canal lengths prior to a confirmatory working length radiograph [23]. If a working length file appears off-centre on the radiograph, multiple canals should be suspected.

Yang et al. [24] reported the distance between the apical foramen and the anatomic apex of most mandibular first premolars to be 0–2 mm. However, larger discrepancies of 2–5 mm were also noted. This finding would definitely need to be borne in mind while confirming the working length of premolars using only radiographic images. In different anatomic studies, the mean length of the mandibular premolars has been reported to be 21.2 [25], 22.4 mm [26], and 21.6 mm [27]. This range of lengths could be used as a guideline, but ethnic variations should be given due consideration.

#### 3.2.2. Biomechanical Preparation

An important aspect that needs to be considered during instrumentation of multiple canals in mandibular premolars is the presence of a sharp curve especially in the bifurcation or trifurcation area (Figure 9). Caution should be exercised during instrumentation along these sharp angles to prevent file separation. Preventive methods include the use of fresh instruments, frequent inspection of files for distortion, precurving hand files, and use of flexible Ni-Ti rotary and hand files. Any incidence of file separation within the complex canal system makes retrieval extremely difficult and also renders the root walls highly susceptible to perforations.

**Figure 12:**
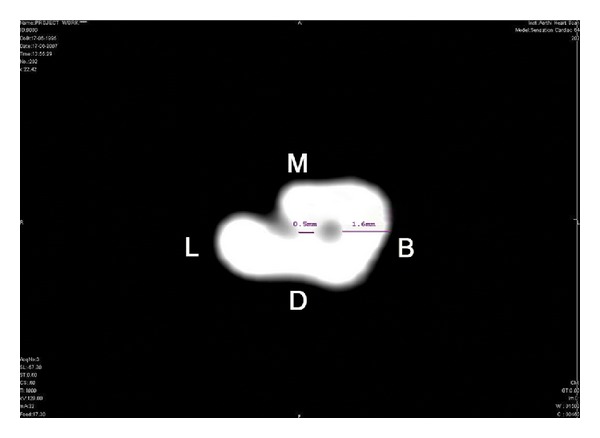
Cross-sectional view at the middle third of a mandibular first premolar tooth showing reduced root dentin thickness (0.5 mm) in relation to the mesiolingual invagination compared to a much wider root thickness of 1.6 mm, buccally. (M) Mesial surface, (D) distal surface, (B) buccal surface, and (L) lingual surface (reprinted with permission from Sandhya et al. [27]).

Prior to commencing the mechanical instrumentation of accessory roots and root canals, it is mandatory to identify and evaluate the morphological features including the thickness of the root dentine. Sandhya et al. [27] reported excessive thinning of root dentin in deep developmental grooves, up to 0.8, 0.78, and 0.3 mm, respectively, in the coronal, middle, and apical thirds. Similar grooves have been observed in other studies as well as in C-shaped canalled roots [15, 26–28]. Such minimal thickness of root dentin could certainly render teeth more vulnerable to failure from treatment procedures (Figure 12). Hence, the clinician should be careful in instrumentation of the mesial surface of the canal, as overzealous instrumentation can lead to strip or lateral perforations in these critical areas. Similarly, caution is warranted if warm root filling or compaction techniques are to be used, as these require more dentine removal for adequate accommodation of heat carriers, delivery needles, and pluggers. Furthermore, the amount of heat transferred through thin dentin walls to the external root surface could cause undesirable thermal damage to the supporting periradicular tissues [29]. The coronal tooth structure also needs to be preserved and widened with caution to avoid complications of postendodontic restorations. Preoperative CBCT images can provide accurate information in these cases regarding the critical remaining dentin thickness in premolars with deep radicular invaginations.

#### 3.2.3. Dynamic Irrigation

In addition to the multiplicity of canals, the pulpal space of mandibular premolars also commonly contains isthmuses, lateral canals, apical ramifications, and other irregularities [30]. The use of adjuncts to cleaning and shaping in the form of passive ultrasonic irrigation, apical negative pressure irrigation devices, or other dynamic modes of irrigation could be useful in enhancing disinfection and cleaning the uninstrumented apical bi- and trifurcations and lateral ramifications that are the most difficult to instrument [30]. The use of an irrigation needle with a smaller diameter could result in enhanced irrigant flow into root canal irregularities [5].

One aspect that should not be overlooked in such cases is the best possible removal of calcium hydroxide intracanal medicament. Any calcium hydroxide remnants may interact with the sealer and result in an inferior seal [31]. Removal of intracanal medicaments with ultrasonic activation of EDTA and NaOCl or irrigating with citric acid has been proposed [5, 32, 33].

#### 3.2.4. Obturation

Root/root canal bifurcation or trifurcation can occur at the cervical, middle, or apical thirds of mandibular premolars. Obturation of these complex root canals may require technique modifications. 

*(i) Cervical Third Bi- or Trifurcation.* Most of the cases can be managed using warm vertical compaction with small sized heat carriers, pluggers, and narrow back filling needles. However, if the root canals are very thin, lateral compaction with thin spreaders or single cone obturation with 6% taper cones is recommended [34]. 

**Figure 13:**
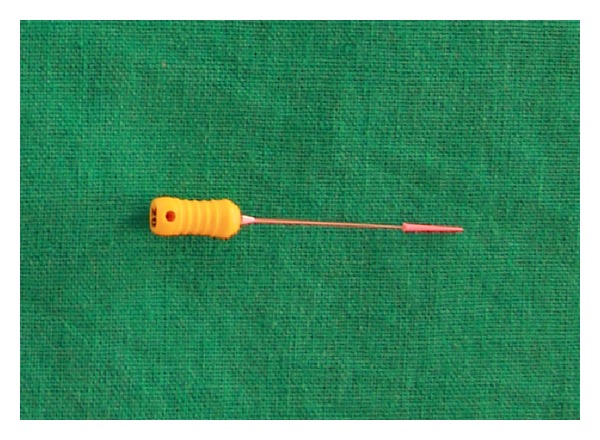
The cut end of a master cone gutta percha pierced with a number 20 spreader.

*(ii) Middle Third Bi- or Trifurcation.* Enhanced magnification and illumination along with adequate coronal canal flaring to access the bifurcated canal orifices are recommended for this technique. When the coronal space is insufficient to accommodate multiple master cones at once, the larger master cone can be cut extraorally, at the level of the bifurcation or orifice. This shortened master cone can then be speared at the blunt, cut end with a spreader or a file in a clockwise rotation (Figure 13). The gutta percha cone is then introduced into the canal, and rotating the file counterclockwise, using apical pressure, loosens the file. A plugger can then be used to compact the gutta percha. Care must be taken to prevent blocking of the unfilled canals by sealer or remnant gutta percha by placing a paper point or a hand instrument within the canal that is to be filled later. The remaining canals can then be obturated similarly. After radiographic confirmation, backfilling of the coronal portion of the canal is done [35]. 

*(iii) Apical Third Bi- or Trifurcation.* Obturating such root canal systems requires good clinical skill along with a dental operating microscope with higher depth of focus. One technique proposed by Hermann and Hülsmann is called the “Squirt technique” [35, 36]. In this technique, both ends of the root canals are obturated simultaneously by injection of thermoplasticized gutta percha, followed by back filling. Alternately, two or three separate master cones are used with each cone being sheared apical to the bi- or trifurcation, allowing room for the next master cone to be seated. Once the individual canals in the apical third are filled, the coronal canal is backfilled.

**Figure 14:**
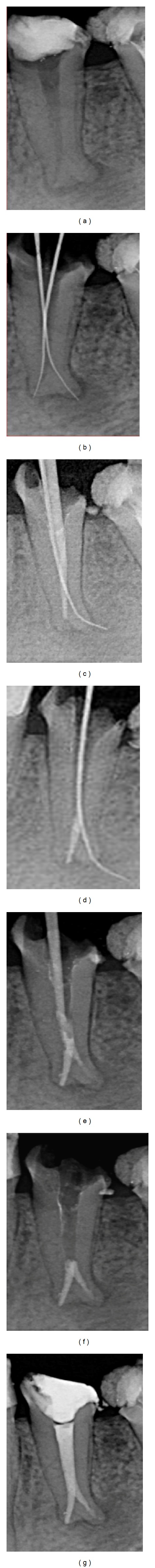
(a) Mandibular premolar with an apical third bifurcation, (b) working length radiograph, (c) an ISO 20 K-file is placed in the mesial canal and the master cone in the distal canal, (d) obturation of the distal canal by warm vertical compaction up to the level of the bifurcation, with the file in mesial canal to prevent its blockage, (e) master cone in the mesial canal, (f) warm vertical compaction of mesial and distal canals, and (g) postobturation radiograph.

Maintaining patency of the off-shoot canal is of prime importance. For this purpose, a file could be placed in one of the canals to prevent its blockage while the other is obturated up to the level of the bi-or tri-furcation by warm vertical compaction (Figure 14). The additional canals are then filled similarly followed by coronal bulk filling.

### 3.3. Surgical Management of Aberrant Premolar Morphology

Surgical endodontics of anomalous mandibular premolars could be performed in conjunction with orthograde root canal treatment, especially for complex apical trifurcations or C-shaped canals. Careful examination of the resected root-end for circumferential openings, using the dental operating microscope, is critical. This allows for identification of any apical third variations and their subsequent retrograde management. A higher incidence of apical ramifications in the apical 3–6 mm of C-shaped Mongoloid mandibular premolars was reported by Lu et al. [15]. In such cases, root-end resection at lengths greater than the common landmark of 3 mm was suggested for a successful clinical outcome.

## 4. Prognosis

Mandibular premolars, because of their complex canal systems, are often considered the most difficult of all teeth on which to perform successful endodontic treatment [3, 4]. According to a Washington study, mandibular first premolars had the highest failure rate of 11.45%. Notably, there was a wide discrepancy in the failure rates among mandibular premolars, with only 4.54% accounting for the mandibular second premolar failures [16]. The higher incidence of complex canal anatomy of the first premolars could account for their higher failure. However, it is essential to note that a long era has passed since these reports for failure rates were reported in mandibular premolars. Various innovations in diagnostics, magnification, operative instruments, and techniques and an updated knowledge with regard to the anatomy of mandibular premolars could certainly improve the endodontic success rates of even the most challenging cases.

## 5. Conclusions

Clinicians undertaking treatment of such complex anatomy cases need to be extremely patient as prolonged and multiple appointments are very much a certainty. The time involved in treating such complex cases of variable tooth anatomy is mainly dependent on the clinical skill, expertise, and proficiency of the endodontist and the armamentarium available to achieve optimum clinical outcome.

Practitioners who regularly treat different population origins should be aware of racial differences and their influence on pulp space anatomy. The risk of missing anatomy during root canal treatments is high due to the complexity of the root canal system in mandibular premolars. Hence, a thorough understanding of normal anatomy and common variations, careful interpretation of angled radiographs, use of three-dimensional imaging, proper access cavity preparation, and a detailed exploration of the interior of the tooth, ideally under magnification, followed by adequate cleaning, shaping, and obturation could collectively play a vital role to ensure endodontic success.

## References

[1] Dowd F (2007). *Mosby's Review for the NBDE Part II*.

[2] Kottoor J, Albuquerque D, Velmurugan N, Kuruvilla J (2013). Root anatomy and root canal configuration of human permanent mandibular premolars: a systematic review. *Anatomy Research International*.

[3] Sert S, Bayirli GS (2004). Evaluation of the root canal configurations of the mandibular and maxillary permanent teeth by gender in the Turkish population. *Journal of Endodontics*.

[4] Slowey RR (1979). Root canal anatomy. Road map to successful endodontics. *Dental Clinics of North America*.

[5] Nallapati S (2005). Three canal mandibular first and second premolars: a treatment approach. *Journal of Endodontics*.

[6] Rajesh Ebenezar AV, Venkatesh A, Vinita Mary A (2013). An unusual occurrence of bilaterally geminated mandibular second premolars resulting in premolar molarization: a case report. *Journal of Conservative Dentistry*.

[7] England MC, Hartwell GR, Lance JR (1991). Detection and treatment of multiple canals in mandibular premolars. *Journal of Endodontics*.

[8] Martínez-Lozano MA, Forner-Navarro L, Sánchez-Cortés JL (1999). Analysis of radiologic factors in determining premolar root canal systems. *Oral Surgery, Oral Medicine, Oral Pathology, Oral Radiology, and Endodontics*.

[9] Kottoor J, Velmurugan N, Surendran S (2011). Endodontic management of a maxillary first molar with eight root canal systems evaluated using cone-beam computed tomography scanning: a case report. *Journal of Endodontics*.

[10] Krithikadatta J, Kottoor J, Karumaran CS, Rajan G (2010). Mandibular first molar having an unusual mesial root canal morphology with contradictory cone-beam computed tomography findings: a case report. *Journal of Endodontics*.

[11] Yu X, Guo B, Li KZ (2012). Cone-beam computed tomography study of root and canal morphology of mandibular premolars in a western Chinese population. *BMC Medical Imaging*.

[12] Al-Fouzan KS (2001). The microscopic diagnosis and treatment of a mandibular second premolar with four canals. *International Endodontic Journal*.

[13] Hülsmann M (1990). Mandibular first premolar with three root canals.. *Endodontics and dental Traumatology*.

[14] Fan B, Yang J, Gutmann JL, Fan M (2008). Root canal systems in mandibular first premolars with C-shaped root configurations. Part I. Microcomputed tomography mapping of the radicular groove and associated root canal cross-sections. *Journal of Endodontics*.

[15] Lu TY, Yang SF, Pai SF (2006). Complicated root canal morphology of mandibular first premolar in a Chinese population using the cross section method. *Journal of Endodontics*.

[16] Ingle JI, Bakland LK, Baumgartner JC (2007). *Ingle's Endodontics*.

[17] Vertucci FJ, Gegauff A (1979). Root canal morphology of the maxillary first premolar. *The Journal of the American Dental Association*.

[18] Ordinola-Zapata R, Bramante CM, Villas-Boas MH, Cavenago BC, Duarte MH, Versiani MA (2013). Morphologic micro-computed tomography analysis of mandibular premolars with three root canals. *Journal of Endodontics*.

[19] Rödig T, Hülsmann M (2003). Diagnosis and root canal treatment of a mandibular second premolar with three root canals. *International Endodontic Journal*.

[20] Tzanetakis GN, Lagoudakos TA, Kontakiotis EG (2007). Endodontic treatment of a mandibular second premolar with four canals using operating microscope. *Journal of Endodontics*.

[21] Krasner P, Rankow HJ (2004). Anatomy of the pulp-chamber floor. *Journal of Endodontics*.

[22] Green D (1973). Double canals in single roots. *Oral Surgery Oral Medicine and Oral Pathology*.

[23] Brunton PA, Abdeen D, MacFarlane TV (2002). The effect of an apex locator on exposure to radiation during endodontic therapy. *Journal of Endodontics*.

[24] Yang H, Tian C, Li G, Yang L, Han X, Wang Y (2013). A cone-beam computed tomography study of the root canal morphology of mandibular first premolars and the location of root canal orifices and apical foramina in a Chinese subpopulation.. *Journal of Endodontics*.

[25] Jain A, Bahuguna R (2011). Root canal morphology of mandibular first premolar in a gujarati population—an in vitro study. *Dental Research Journal*.

[26] Awawdeh LA, Al-Qudah AA (2008). Root form and canal morphology of mandibular premolars in a Jordanian population. *International Endodontic Journal*.

[27] Sandhya R, Velmurugan N, Kandaswamy D (2010). Assessment of root canal morphology of mandibular first premolars in the Indian population using spiral computed tomography: an in vitro study. *Indian Journal of Dental Research*.

[28] Robinson S, Czerny C, Gahleitner A, Bernhart T, Kainberger FM (2002). Dental CT evaluation of mandibular first premolar root configurations and canal variations. *Oral Surgery, Oral Medicine, Oral Pathology, Oral Radiology, and Endodontics*.

[29] Lipski M (2005). Root surface temperature rises during root canal obturation, in vitro, by the continuous wave of condensation technique using System B HeatSource. *Oral Surgery, Oral Medicine, Oral Pathology, Oral Radiology and Endodontology*.

[30] Peters OA (2004). Current challenges and concepts in the preparation of root canal systems: a review. *Journal of Endodontics*.

[31] Margelos J, Eliades G, Verdelis C, Palaghias G (1997). Interaction of calcium hydroxide with zinc oxide-eugenol type sealers: a potential clinical problem. *Journal of Endodontics*.

[32] Boutsioukis C, Psimma Z, Kastrinakis E (2014). The effect of flow rate and agitation technique on irrigant extrusion ex vivo. *International Endodontic Journal*.

[33] Rödig T, Vogel S, Zapf A, Hülsmann M (2010). Efficacy of different irrigants in the removal of calcium hydroxide from root canals. *International Endodontic Journal*.

[34] Whitworth J (2005). Methods of filling root canals: principles and practices. *Endodontic Topics*.

[35] Hermann HW, Hülsmann M, Hülsmann M, Schäfer E (2009). Problems of root canal obturation. *Problems in Endodontics—Etiology, Diagnosis and Treatment*.

[36] da Silva D, Endal U, Reynaud A, Portenier I, Ørstavik D, Haapasalo M (2002). A comparative study of lateral condensation, heat-softened gutta-percha, and a modified master cone heat-softened backfilling technique. *International Endodontic Journal*.

